# One Health Approach: A Data-Driven Priority for Mitigating Outbreaks of Emerging and Re-Emerging Zoonotic Infectious Diseases

**DOI:** 10.3390/tropicalmed7010004

**Published:** 2021-12-29

**Authors:** Busayo I. Ajuwon, Katrina Roper, Alice Richardson, Brett A. Lidbury

**Affiliations:** 1 National Centre for Epidemiology and Population Health, Research School of Population Health, ANU College of Health and Medicine, The Australian National University, Acton, ACT 2601, Australia; katrina.roper@anu.edu.au (K.R.); brett.lidbury@anu.edu.au (B.A.L.); 2 Department of Microbiology, Faculty of Pure and Applied Sciences, Kwara State University, Malete 241103, Nigeria; 3 Statistical Support Network, The Australian National University, Acton, ACT 2601, Australia; alice.richardson@anu.edu.au

**Keywords:** One Health, infectious disease, surveillance systems, global health security, machine learning capability

## Abstract

This paper discusses the contributions that One Health principles can make in improving global response to zoonotic infectious disease. We highlight some key benefits of taking a One Health approach to a range of complex infectious disease problems that have defied a more traditional sectoral approach, as well as public health policy and practice, where gaps in surveillance systems need to be addressed. The historical examples demonstrate the scope of One Health, partly from an Australian perspective, but also with an international flavour, and illustrate innovative approaches and outcomes with the types of collaborative partnerships that are required.

## 1. Introduction

Recurring outbreaks of emerging and re-emerging zoonotic infectious diseases constitute a significant threat to global health security. Progress towards global health security requires a greater focus on One Health concept. This concept recognises the interconnectedness of humans, animals, and their shared environment, and a strong collaboration between multiple sectors for better public health outcomes [1]. In December 2019, the zoonotic coronavirus, named severe acute respiratory syndrome coronavirus 2 (SARS-CoV-2), caused large-scale outbreaks in Wuhan, China [2]. The pathogen, which was speculated to have emerged from its ancestral bat [3], has since spread worldwide, recording 255,324,963 cases with 5,127,696 deaths as of 4:50pm CET, 19 November 2021 [2]. In addition to becoming a major global health threat, the 2019 novel coronavirus disease (COVID-19) has provoked significant economic and diplomatic fallout. Consequently, it has become compelling to halt the continued emergence of this virus using a One Health approach, particularly with recent reports from the Netherlands, Italy, Spain, the USA, Sweden, Greece, and, more recently, Denmark [4,5], all suggesting possible spillover. SARS-CoV-2 can change while infecting minks. It has been observed that these mink variants are able to transmit back into humans through close contact with the mink.

Hammer et al. [6], in the study conducted in Denmark, reported SARS-CoV-2 variants in farmed mink using genomic sequence analysis. These diverse sequences of variants, subsequently identified within the local human community, showed a combination of new mutations that have not been previously investigated. The genome sequencing evidence of the mink-associated variant strain has reaffirmed the transmission risks to persons and reinforced the inextricable links between humans, animals, and the environment. This raises a major concern about the potential establishment of a SARS-CoV-2 non-human reservoir, from where the virus could be reintroduced once the human circulation is halted. Although, there is currently no evidence of SARS-CoV-2 spillover from other infected animals to human, outside of farmed minks. However, the already established bi-directional nature of SARS-CoV-2 transmission potentially constitutes a permanent pandemic threat, especially if One Health capabilities are not implemented as a priority for interventions, in and amongst the nations of the world.

The frequency of the re-emergence of infectious diseases from animal origins, such as SARS and MERS coronaviruses, the avian influenza viruses, and Ebola viruses, is unprecedented and is expected to increase due to the complex inter-connecting relationship between humans, animals, and the environment. Human activities, including intensive farming practices, ecosystem and land-use changes, urbanisation, and international travel and trade, are principal factors that drive the re-emergence of zoonotic infectious diseases. In order to understand the ecology of emerging zoonotic diseases and support countries in achieving sustainable and functional collaboration at the human-animal-environment interface, the Food and Agriculture Organisation of the UN, the World Health Organisation, and the World Health Organisation for Animal Health consolidated a formal partnership to combat health-animal-environment health risks. This strategic partnership led to the formal launch of a tripartite guide to addressing zoonotic diseases, using a multisectoral, One Health approach [7].

Comparative medicine has long been acknowledged for its numerous innovative benefits in sciences, and One Health expands the scope of comparative medicine to surveillance in animals and the environment for early disease detection and better understanding of threats to mitigate risk. For example, die-offs of great apes associated with Ebola virus have often been detected prior to outbreaks in humans, providing a potential predictive value that can help prevent human cases, if paired with risk mitigation measures, such as hunter avoidance of carcasses [8]. Weather conditions have also been used to forecast Rift Valley fever and other outbreaks, and can inform vaccination and mosquito control campaigns to reduce the health and economic consequences of disease epidemics [9]. Further, the onset of encephalitis cases in people and birds, which were ultimately linked to the emergence of West Nile virus in the U.S. in 1999, challenged public health authorities in identifying its origin. However, critical insight into the cause of disease was gained from a collaborative investigation with the veterinary community of associated wild bird mortalities. Sentinel surveillance in mosquitoes, birds, and horses is now routinely being used to monitor risk to human health and trigger preventive measures [10].

The management of SARS outbreak in early 2003 and the spread of highly pathogenic avian influenza are historical examples that further demonstrate the power of One Health as well as the dangers of not implementing effective mechanisms between and within countries for its sustainability. For both the SARS outbreak and the avian influenza, the One Health approach was a key paradigm in stemming the ramifications of these diseases and preventing a pandemic. For example, following the 2017 avian influenza outbreak in neighbouring Uganda, the Rwanda Agricultural Board, in collaboration with the National Health Steering Committee, conducted a field investigation of an avian mortality event in Rwanda and organised a community risk sensitization, despite not having any confirmed cases. This collaborative effort strengthened Rwanda’s preparedness and improved their national contingency plan against the highly pathogenic avian influenza. Timely implementation of robust integrated disease surveillance and monitoring, urban planning, and health infrastructure, all of which coalesce in One Health, would have quashed the worldwide spread and reduced the threats posed to global health security. Another key milestone in One Health evolution was the 2008 Hendra virus (HeV) outbreak in Australia, which involved multiple equine and confirmed human cases at a veterinary clinic in Brisbane, Queensland [11]. Subsequent to this outbreak, an inter-agency technical working group was constituted to provide evidence-based, science-driven, best practice recommendations to stem the transmission of this virus, and inform joint animal and public health policy. The inter-agency collaboration revealed the strength of One Health and championed the evolution of the One Health approach to HeV in Australia. Australia’s One Health approach to controlling the spread of HeV led to an informed, effective, and efficient management of what could have been a very complex political, social, and biological system. Other added values of One Health, which might have been impossible to achieve if human and animal health sectors had worked independently, have been reported and are summarised in Table 1.

In September 1998, the Nipah virus outbreak in Malaysia (originally thought to be Japanese encephalitis virus) resulted in 265 cases of acute encephalitis with 105 deaths due to the ineffectiveness of the early control measures, which led to near collapse of the billion-dollar swine industry [17]. The Nipah epidemic ensued from a chain of infection, initiating from bats to pigs, and subsequently migrated from pigs to humans [18]. Several factors, including inadequate surveillance and ecological modelling of the disease transmission; lack of an open-minded approach and co-ordination between medical scientists, veterinarians, and wildlife specialists; and environmental mismanagement, contributed to gaps in the strategies implemented to control the early phase of the outbreak which raged through the country for 6 months [19].

A One Health approach is critical to address health threats at the interface of humans, animals, and the environment [1]. Establishing sustainable collaboration across all sectors and disciplines at the country level allows countries to effectively prepare for, detect, assess, and respond to emerging and re-emerging zoonotic diseases as well as other shared health threats. In settings where there is a lack of coordinated planning and established mechanisms for collaboration, public health stakeholders cannot implement a timely and effective response to disease events and emergencies. National mechanisms for adopting a multisectoral, One Health approach for zoonotic diseases are rarely available, but the lack of basic mechanisms for implementing effective inter-ministerial collaboration can obstruct the implementation of effective disease control programmes and lead to poorer health outcomes, especially in resource-limited countries. In most countries, a preparedness and surveillance plan for zoonotic diseases is often developed by, and for, a single sector. A single sector’s strategy leads to disjointed activities, lack of information sharing engagement, and an inability to provide adequate emergency preparedness and response.

## 2. Bolstering Surveillance System Using Data-Driven One Health Approach

A One Health approach can be invaluable for establishing and improving local, national, and global surveillance for early detection of zoonotic disease events and facilitating prompt sharing of data for a coordinated response. The benefits of taking a multisectoral One Health approach to zoonotic diseases include strengthening systems and coordination across the human health, animal health and the environment, improving the efficiency and effectiveness of disease management, which can reduce cost, supporting outbreak investigation and response, and facilitating evidence-based decision making for improved public health outcomes [1]. One Health approach provides mechanisms for additional activities such as operational emergency responses, and prompt reporting to national and international responses. A country on its own has limited capabilities, particularly when responding to a new threat; thus, a shared responsibility approach could support the management of any complex health issue, and be implemented at different levels and settings. For example, knowledge needs to be shared about disease epidemiology, effective treatments, people’s reactions to preventive measures and testing protocols, amongst others.

**Figure 1:**
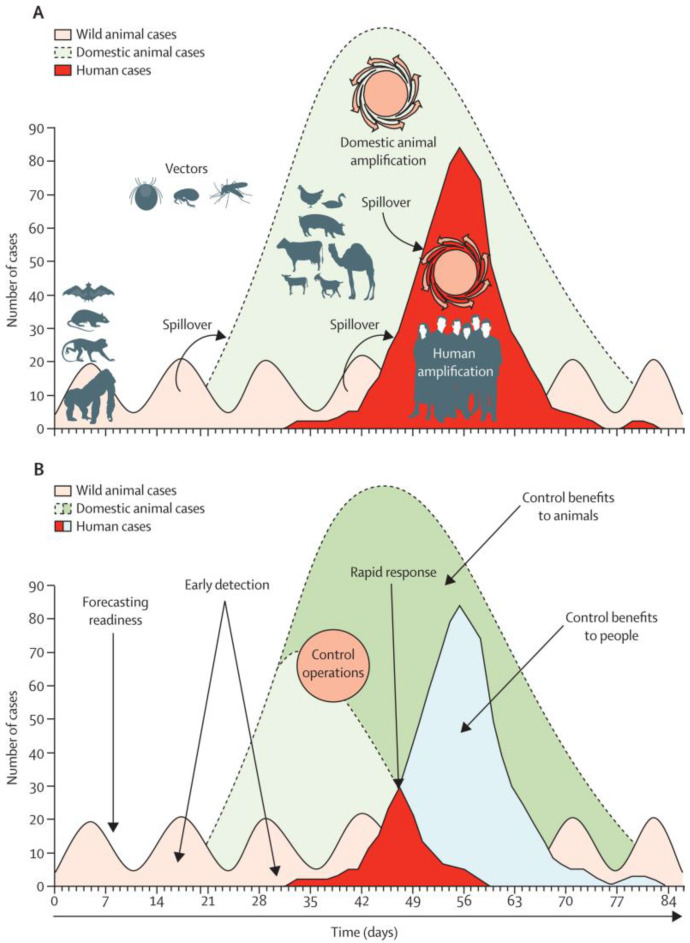
Clinical relevance of disease ecology. Source: Adapted from Karesh et al. [20]. (**A**) Transmission of infection and amplification in people (bright red) occurs after a pathogen from wild animals (pink) moves into livestock to cause an outbreak (light green) that amplifies the capacity for pathogen transmission to people. (**B**) Early detection and control efforts reduce disease incidence in people (light blue) and animals (dark green). Spillover arrows show cross-species transmission.

Further, a surveillance system for zoonotic diseases, coordinated through a data-driven One Health approach, includes machine learning modelling capability. Coupled with data variables from preparedness, investigation, and response, a coordinated One Health surveillance system can be used to project disease progression and predict as accurately as possible the magnitude and time of a potential outbreak, based on a range of mathematical model assumptions and an understanding of pathogen transmission dynamics. An understanding of the relationship between environmental changes, wildlife population dynamics, and the dynamics of their microorganisms can be used to understand the risk of human infection with endemic zoonoses [20] (Figure 1). For example, Mollentze et al. [21], in a recent study, developed machine learning models that use viral and human genome sequence features to predict the probability that an animal virus might jump into humans. The machine learning model, which correctly identified 70.8% of human viruses with high or very high zoonotic potential, demonstrates the potential of machine learning modelling capability in determining the risk of a viral spillover from animals to humans.

There will be benefits to mapping all the existing coordinating mechanisms and other aspects of the zoonotic disease surveillance system for each country. The data provided by mapping can identify overlaps, gaps, and synergies among the relevant stakeholders’ activities. The identified gaps and overlaps can then be addressed by building networks and partnerships within and between countries to standardise the surveillance system’s design and strengthen its sensitivity for detecting new or unusual events. These partnerships should include all relevant disciplines and sectors, such as public health, animal health, environment sectors, universities, and international and community partners, all of which should communicate regularly and adopt a multisectoral approach to collaboration. For example, laboratories within a country’s surveillance system should standardise diagnostic techniques and align local procedures with internationally recognised standards.

Ensuring equitable financing among relevant sectors is critical for the sustainability of a system that reduces risks from zoonotic diseases. Existing studies have shown that reduced risks from zoonotic diseases also reduce indirect societal losses, such as poorer nutrition and restriction of tourism and trade, which, if included, would bring the global costs of recent zoonotic disease events to tens of billions of dollars [1,22]. Data on the sources of financing and cost projections associated with the implementation of One Health are limited, particularly from countries with limited infrastructure. Consequently, research on cost-benefit analyses of One Health would be beneficial for engaging stakeholders and helping policy makers understand how costs and benefits are shared across sectors. 

## 3. Conclusions

Strengthening systems and coordination across the human, animal, and environment interface can save costs by preventing duplication of activities and providing a positive return on investment. For example, Guinea, Sierra Leone, and Liberia were projected to suffer a crippling loss in economic growth in 2015, as a result of the Ebola epidemic. After the three countries had initially recorded positive economic growth in 2013, their GDP growth declined post-Ebola epidemic, with Sierra Leone’s GDP dropping sharply from 4.6% in 2014 to −12.5% in 2015 [23]. These economic losses could have been prevented, if a more resilient and integrated health system was in place. Establishing a strong integrated system, which specifically collates data about ecosystems and incorporates primary data collection on disease parameters from people, livestock, and wildlife, would help to better clarify the dynamics of emerging and re-emerging infectious diseases and response. The application of big-data-driven machine learning algorithms and classical statistical methods, to such One Health data, would allow for newer capabilities that complement traditional disease surveillance systems both in situ and in vivo. Such analytically driven approaches would provide insight into the burden of zoonoses and help investigate patterns that can predict potential outbreaks at a much lower economic cost than what is required for response after the pathogens might have emerged.

## Figures and Tables

**Table 1:** Added value of One Health.

Domain	Added Value	Reference
Health services	Access to health care through joint human and animal vaccination services for mobile pastoralists	Schelling et al. [12]
Zoonoses control	Public health benefits from mass vaccination of livestock against brucellosis	Mindekem et al. [13]
Surveillance and response	Returns of over €1 million in savings through an integrated surveillance and response to West Nile virus	Paternoster et al. [14]
Infrastructure	26% savings in operational cost for hosting national human and animal health laboratories under one roof	World-Bank [15]
Communication	Strengthened health systems through sustained and targeted communication of the science involved	Enserink [16]

## Data Availability

Not applicable.
